# Study on the clinical application of pulsed DC magnetic technology for tracking of intraoperative head motion during frameless stereotaxy

**DOI:** 10.1186/1746-160X-2-10

**Published:** 2006-04-26

**Authors:** Olaf Suess, Silke Suess, Sven Mularski, Björn Kühn, Thomas Picht, Stefanie Hammersen, Rüdiger Stendel, Mario Brock, Theodoros Kombos

**Affiliations:** 1 Department of Neurosurgery, Charité – Universitaetsmedizin Berlin, Campus Benjamin Franklin, Berlin, Germany

## Abstract

**Background:**

Tracking of post-registration head motion is one of the major problems in frameless stereotaxy. Various attempts in detecting and compensating for this phenomenon rely on a fixed reference device rigidly attached to the patient's head. However, most of such reference tools are either based on an invasive fixation technique or have physical limitations which allow mobility of the head only in a restricted range of motion after completion of the registration procedure.

**Methods:**

A new sensor-based reference tool, the so-called Dynamic Reference Frame (DRF) which is designed to allow an unrestricted, 360° range of motion for the intraoperative use in pulsed DC magnetic navigation was tested in 40 patients. Different methods of non-invasive attachment dependent on the clinical need and type of procedure, as well as the resulting accuracies in the clinical application have been analyzed.

**Results:**

Apart from conventional, completely rigid immobilization of the head (type A), four additional modes of head fixation and attachment of the DRF were distinguished on clinical grounds: type B1 = pin fixation plus oral DRF attachment; type B2 = pin fixation plus retroauricular DRF attachment; type C1 = free head positioning with oral DRF; and type C2 = free head positioning with retroauricular DRF. Mean fiducial registration errors (FRE) were as follows: type A interventions = 1.51 mm, B1 = 1.56 mm, B2 = 1.54 mm, C1 = 1.73 mm, and C2 = 1.75 mm. The mean position errors determined at the end of the intervention as a measure of application accuracy were: 1.45 mm in type A interventions, 1.26 mm in type B1, 1.44 mm in type B2, 1.86 mm in type C1, and 1.68 mm in type C2.

**Conclusion:**

Rigid head immobilization guarantees most reliable accuracy in various types of frameless stereotaxy. The use of an additional DRF, however, increases the application scope of frameless stereotaxy to include e.g. procedures in which rigid pin fixation of the cranium is not required or desired. Thus, continuous tracking of head motion allows highly flexible variation of the surgical strategy including intraoperative repositioning of the patient without impairment of navigational accuracy as it ensures automatic correction of spatial distortion. With a dental cast for oral attachment and the alternative option of non-invasive retroauricular attachment, flexibility in the clinical use of the DRF is ensured.

## Background

Imaging of the intracranial anatomy with direct visualization of a pathological lesion became possible only with the advent of computer-based imaging modalities in the 1970s. Roberts et al. [1] were among the first in 1986 to integrate the spatial information on tumour extent as calculated by a computer into the surgical microscope image without using a rigid external reference frame. Only one year later, Watanabe et al. [2] presented a device specifically developed for what is still known as „frameless stereotaxy”. The authors presented a computer-based device that uses a multijointed arm to identify target points predefined in preoperatively acquired images. This enabled both, precise trepanation and corticotomy sparing functionally important cerebral areas and the reliable identification of deeply located small lesions. The investigators referred to the device they had developed as a "neuronavigator" and thereby coined a term that continues to be used for a whole family of devices that serve to precisely determine the spatial position of anatomic structures under difficult and intricate operative conditions.

Various neuronavigation systems were technically perfected in the course of the 1990s. The fact that different groups all over the world developed these devices independently soon led to the use of different physical methods for the highly complex process of integrating image data into the surgical field. Thus, the current neuronavigation market offers not only systems on the basis of image-controlled articulated arms [3] but also camera-based systems [4,5], sonographically [6] or microscopically guided systems [1], and finally systems recording positional information by means of sensors within an electromagnetic field [7,8].

Independently of the system employed, a process called "image data registration", is necessary to match the navigation image dataset and the patient's head position after positioning for the operation. Registration consists in matching a number of reference points on the patient's head (e.g. fiducial markers, landmarks, or surface reliefs) with corresponding points in the preoperatively acquired image datasets using special algorithms [9-11]. The accuracy of this alignment process directly determines the system's overall application accuracy and the accuracy in detecting a circumscribed target in the operating field. This is why most navigation systems in which the tracking system itself serves as reference require rigid fixation of the patient's head during the complete course of the procedure. Such rigid immobilization of the head is typically done using commercially available head clamps with multiple pin fixations.

However, to allow intraoperative re-positioning of the head (like in patients with multilocular lesions or certain skull base procedures) or free head mobility for certain indications (such as burr hole procedures for intracranial endoscopy or biopsies), it has been proposed to track intraoperative head motion in direct relation to the manoeuvres performed with the surgical instruments. This approach relies on a fixed reference device rigidly attached to the patient. Various attempts in detecting and compensating for intraoperative head motion during frameless stereotaxy have already been described. Some of these approaches are based on setups in which an additional reference frame is directly (invasively) attached to the patient's head, such as an additional scalp screw for fixation of the frame [12] or the attachment of a modified reference clamp on the boundary of the craniotomy [13]. Other investigators have described non-invasive techniques of head fixation such as tailored masks [14] and pin-free head holders [15,16], or non-invasive extracorporal reference frames such as specially designed headsets [17] or dental casts for fixation of an additional reference tool [18]. Nevertheless, all of these techniques have physical limitations which allow mobility of the head only in a restricted range of motion after completion of the registration procedure. That's why preliminary results with a DC (direct current) magnetic navigation technique for tracking of the patient's head and target motion in frameless stereotaxy [19] have encouraged the authors to test a new sensor-based dynamic reference frame (DRF) which is designed to allow an unrestricted, 360° range of motion for the intraoperative use in cranial neurosurgery. Different methods of non-invasive attachment dependent on the clinical need and indication, as well as the resulting accuracies in the clinical application have been analyzed.

## Methods

### Navigation system

A frameless navigation system (ACCISS II™, Schaerer Mayfield Technologies GmbH, Berlin, Germany) was used for intraoperative image guidance in all cases. The system comprises the hard- and software necessary to generate and detect a DC pulsed magnetic field for computing the position and orientation of a localizing sensor. The tracking system in its basic version consists of an electromagnetic transmitter unit, a sensor (which is integrated into the handle of a surgical pointer device) and an electronic digitizer unit that controls the transmitter and receives the spatial data from the localizing sensor.

The transmitter consists of a triad of electromagnetic coils (size: 9.6 cm cube) which generates a homogeneous electromagnetic field (max. 600 milligauss with a translation range of 76.2 cm in any direction) that, in its basic version, simultaneously serves as the fixed reference for the setup.

The localizing sensors can be integrated into pointers or other surgical instruments of various shapes. The sensor, being completely passive and having no active voltage applied, detects the magnetic field generated by the transmitter unit with up to 120 measurements per second what ensures real-time conditions. They have 6 degrees of freedom (position and orientation) with an angular range of ± 180° azimuth & roll and ± 90° elevation. The static accuracy is specified by the manufacturer (Ascension Technologies Corp., Burlington, USA) as 1.8 mm RMS (position) and 0.5° RMS (orientation). The static resolution is 0.5 mm (position) and 0.1° (orientation) at a distance of 30.5 cm from the transmitter.

In the digitizer unit, the analogue measured signals of the sensor are digitalized, and the coordinates of the sensor position are calculated.

### Dynamic Reference Frame (DRF)

**Figure 1:**
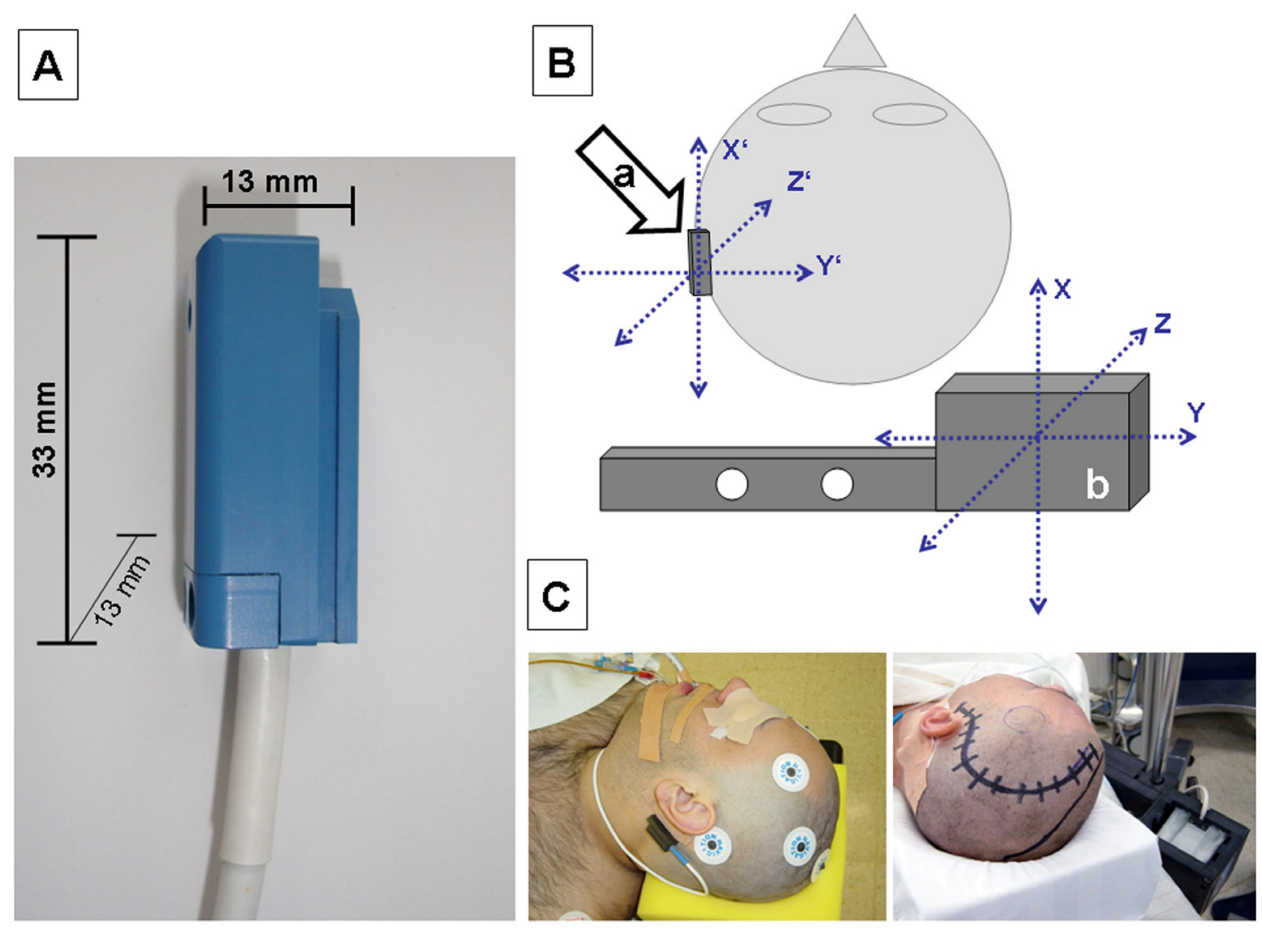
**(A) **Waterproof encapsulated DRF sensor for retroauricular use. **(B) **The DRF (a) can be used as an additional reference system that defines an independent coordinate system in space in addition to the one established by the transmitter unit (b). **(C) **The DRF was placed and fixed with tape draping in direct contact with the back of the auricle.

To allow simultaneous registration, localization and position tracking of more than one localizing sensor, the before described basic version of the ACCISS II system was expanded with a soft- and hardware update which helps to run a so-called Dynamic Reference Frame (DRF). The DRF can be used as an additional reference system that defines an independent coordinate system in space in addition to the one established by the transmitter unit (Figure 1B). Thus, it becomes possible to record the slightest movement of the cranium as well. This information can then be used to continuously adapt the position of the imaging plane and the resultant calculated virtual 3-D model to the actual position of the cranium. Technically, the DRF consists of an additional localizing sensor measuring 8 mm × 8 mm × 18 mm in size with a weight of 1.2 g. The extra sensor is accommodated in a watertight capsule and is connected to the navigation system with a 3 m long cable. The DRF sensor can either (a) be attached to a dental splint or (b) be attached retroauricular on the hairless skin.

**Figure 2:**
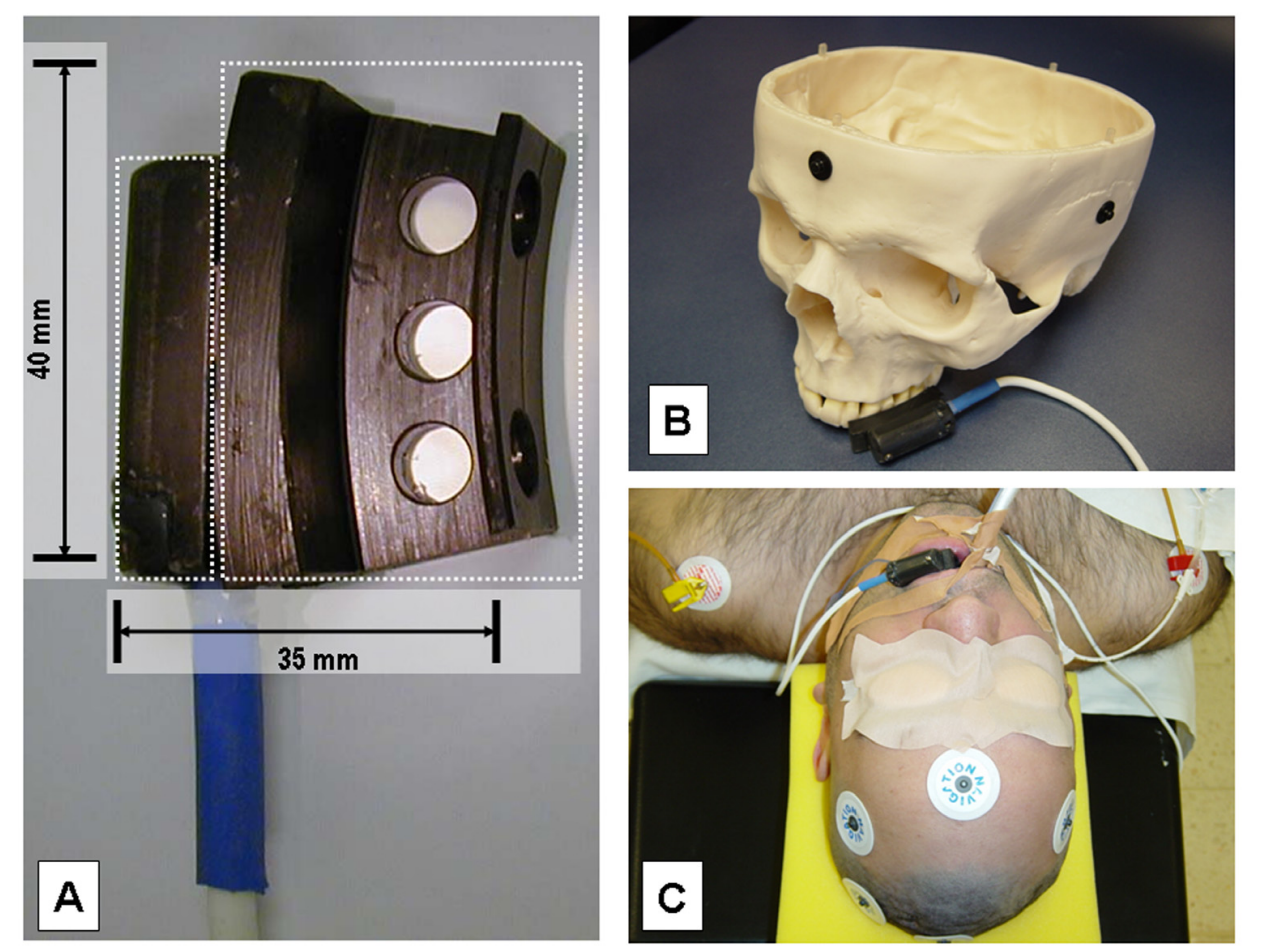
**(A) **DRF with dental cast for the oral use. (B) Example of the fixation technique in a skull dummy and (C) in a patient without rigid head fixation (Type C_1_).

(a) In the oral cavity, the DRF is attached to the upper row of teeth using a special, removable mouthpiece (Figure 2) and a 2-component polyether self-hardening material (Impregum^® ^F; ESPE Dental AG, Seefeld, Germany). The mouthpiece consists of a U-shaped splint which is filled with a fast-hardening material and applied to the upper row of teeth exerting slight pressure (about 0.25 atm) at the centre. The vacuum resulting from hardening of the material ensures that the mouthpiece is firmly secured in place in patients with healthy teeth. After the procedure, the mouthpiece is removed by releasing the vacuum with a dental hook.

Alternatively, if oral attachment is precluded by the patient's dental status or for anesthesiological or surgical reasons, the DRF is attached directly to the scalp, preferably over the mastoid, behind the auricle.

(b) For retroauricular attachment (Figure 1), the DRF is placed in the area of the mastoid in such a way that it is in direct contact with the back of the auricle. The auricle thus serves as an anatomical barrier against anterior displacement. The retroauricular region is chosen because there is minimal skin mobility and the auricle provides additional stability, ensuring stable attachment of the DRF in this area. The device was secured in place with 40 mm wide, skin-friendly tape applied crosswise to the hairless skin (Figure 1C). To prevent detachment of the tape by contact with fluids or disinfectants, a waterproof self-adhesive sterile film was glued over it (Opraflex^®^, Lohmann & Rauscher Int., Rengsdorf, Germany).

**Figure 3:**
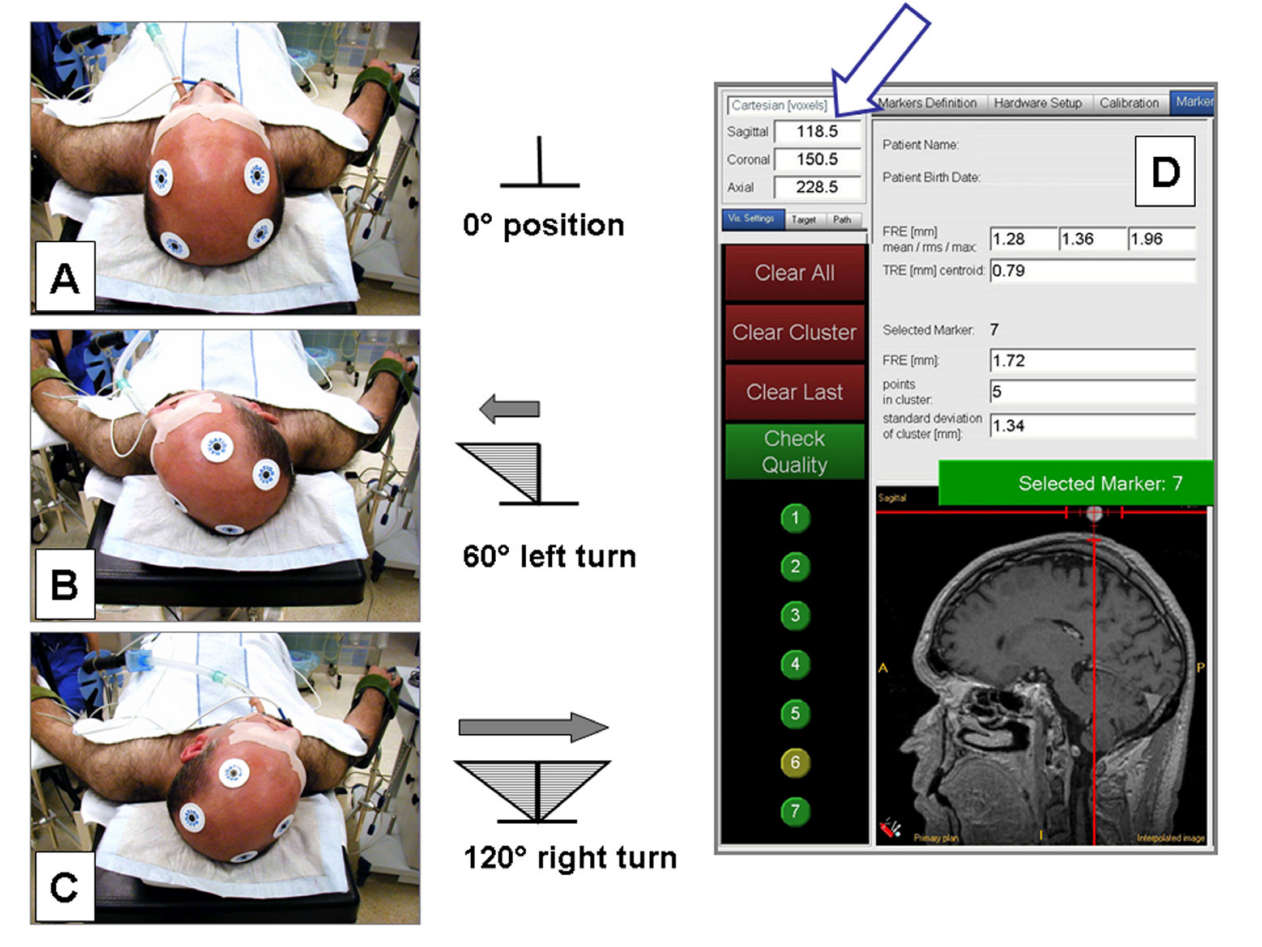
**(A-C) **Proper affixation of the DRF was checked in all cases by a rotation test immediately after image data registration. **(D) **The spatial coordinates (arrow) of the fiducial markers were verified relative to the position of the DRF.

Proper affixation of the DRF was checked in all cases by a rotation test immediately after image data registration (Figure 3). To this end, the head was rotated about 120° from the right lateral to the left lateral position and back (Figure 3A–C). The spatial coordinates of the fiducial markers were verified relative to the position of the DRF. Adequate attachment of the DRF was assumed when the deviation was < 1 mm in all three spatial direction (cartesian x, y, z-coordinates as displayed by the navigation system; Figure 3D). If there was greater deviation, the position was corrected and the attachment optimized until deviation was within the limit of 1 mm.

### Image data acquisition and preparation

Preoperatively, a serial CT or MRI scan was obtained. The images consisted of a three-dimensional volume data set of contiguous axial CT or sagittal MR images. In order to obtain isotropic voxels of 1 mm length one of the following CT or MRI protocols was routinely used.

MRI was performed using a T1-weighted 3D GE sequences (3D MP RAGE) with the parameters: TR 9.7 ms, TE 4 ms, FA 12°, TI 300 ms, TD 0 s, FOV 256 mm, 256 × 256 matrix, 256 partitions, slice thickness of 1 mm, acquisition time 11 min 54 s. Alternatively, a high-resolution CT spiral scan was acquired with 1 mm slice thickness, 512 × 512 matrix, pitch factor 2, 1 mm increment, and 50–110 mA tube current. The image data were transferred to the computer workstation in the ACR/NEMA 3.0/DICOM image data format via a local network (LAN – FTP or DICOM transfer protocol), or through data media, such as CD-ROM, magnetic-optical disks (MOD) or magnetic tape (DAT).

**Figure 4:**
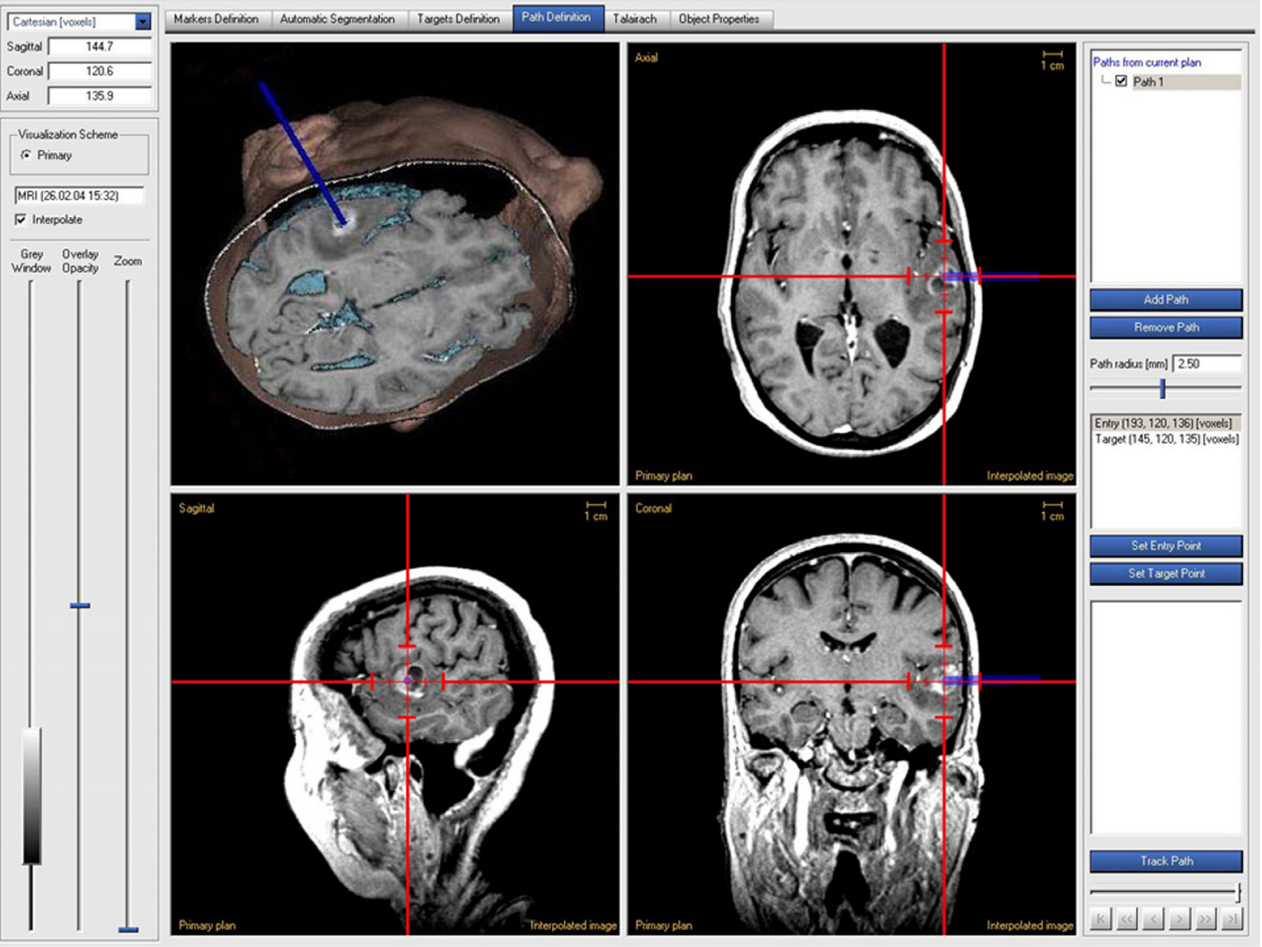
Image guidance was based on axial planar views (sagittal, coronal and transaxial), free planar views, and 3D views of the anatomical objects with tools for targeting and trajectory planning.

Data processing and preparation was performed using an autosegmentation technique (ACCISS II software version 1.9). Image guidance was based on axial planar views (sagittal, coronal and transaxial), free planar views (defined by pointer orientation and/or target localization), and 3D views of the anatomical objects (skin, skull, brain surface structures, brain parenchyma and lesion target) (Figure 4). The image data was registered by means of point-to-point matching (sequentially sampling 7 two-component adhesive fiducial markers with a sensor-bearing pointer according to a standardized protocol).

### Accuracy measurements

*Registration accuracy *was determined calculating the fiducial registration error (FRE) expressed as the root mean square error. The FRE describes the distance between the position of a marker in the image dataset and the position measured in the operative field. The mean RMS value is calculated directly by the navigation system and is displayed together with the min. and max. FRE and the Target Registration Error (TRE – for a certain target point within the registered volume) on the navigation screen (Figure 3D).

**Figure 5:**
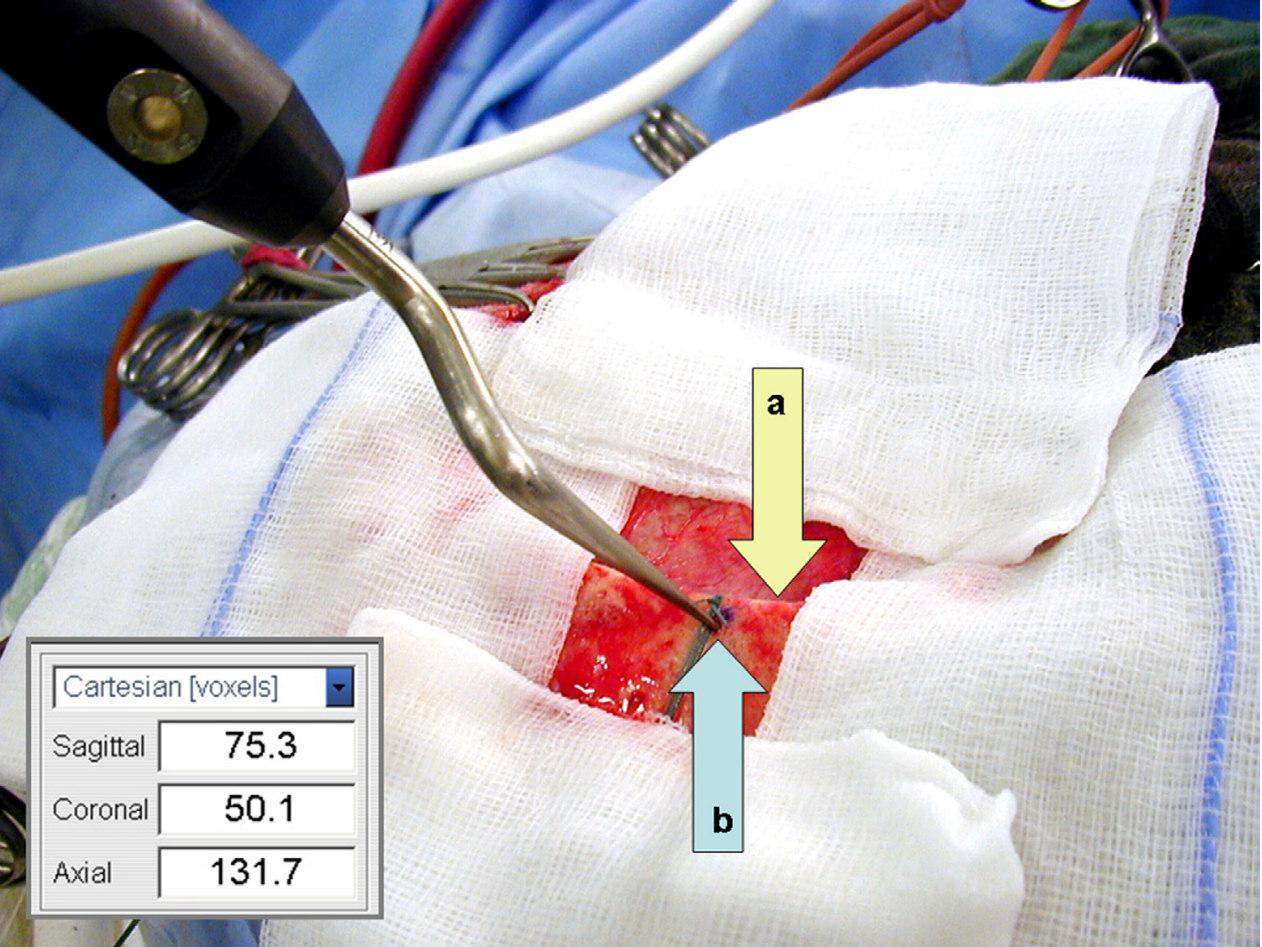
The application accuracy was monitored intraoperatively using as a reference point a 1 mm burr hole (b) drilled into the exposed bone margin (a) directly after craniotomy. The Cartesian coordinates of this reference point were used to calculate the intraoperative Position Error (*PE *= (Δ*sagittal *^2 ^+ Δ*coronal *^2 ^+ Δ*axial *^2^)^1/2^) in mm.

The *application accuracy *was monitored intraoperatively using as a reference point a 1 mm burr hole drilled into the exposed bone margin directly after craniotomy (Figure 5). The initial Cartesian coordinates of this reference point were determined immediately by means of a pointer. The measurements were repeated after craniotomy immediately before dura opening, three times during tumour resection (M1–M3) and after closure of the dura, respectively at the end of the operation. Deviations in x, y, and z directions were measured as three-dimensional Position Error (PE in mm) of the reference point relative to the baseline coordinates of the same point determined immediately after craniotomy.

### Statistical analysis

FRE and PE values are expressed as means +/- standard deviation from the number (*n*) of patients in each group. Data were tested for significance using one-way ANOVA to determine degree of variability within a group, followed by Bonferroni post hoc analysis. Test of pairwise comparisons were carried out with the Student's *t*-test to compare two groups (e.g. for differences in FRE between the different types of head fixation, as well as for differences in ΔPE in-/decrease between the different types of head positioning over the time of surgery). A p < 0.05 was considered as statistically significant. Data management and statistical analyses were performed using the SPSS 13.0 for Windows^® ^software package.

## Results

### Patients and indications for frameless stereotaxy

The clinical study included 40 patients with intracerebral tumours or lesions in the area of the skull base in whom intraoperative navigation was used to localize the target or trajectory or to determine the extent of resection. The patients had the following diagnoses: 2 WHO II gliomas, 8 WHO III gliomas, 7 glioblastomas, 12 metastases, 2 primary bone tumours, 2 meningiomas, 4 lymphomas, 2 fibrous dysplasia, and one paraganglioma. There were 22 men and 18 women with a mean age of 55.7 years (range 18 – 81 years). CT data sets were used for navigation in 9 cases and MRI data sets in the remaining 31 patients. All steps of the examinations were approved by the institutional review board. Written informed consent was available from all patients participating in the study. The interventions were performed at the Department of Neurosurgery, Charité – Universitätsmedizin Berlin, Campus Benjamin Franklin.

### Clinical application

**Figure 6:**
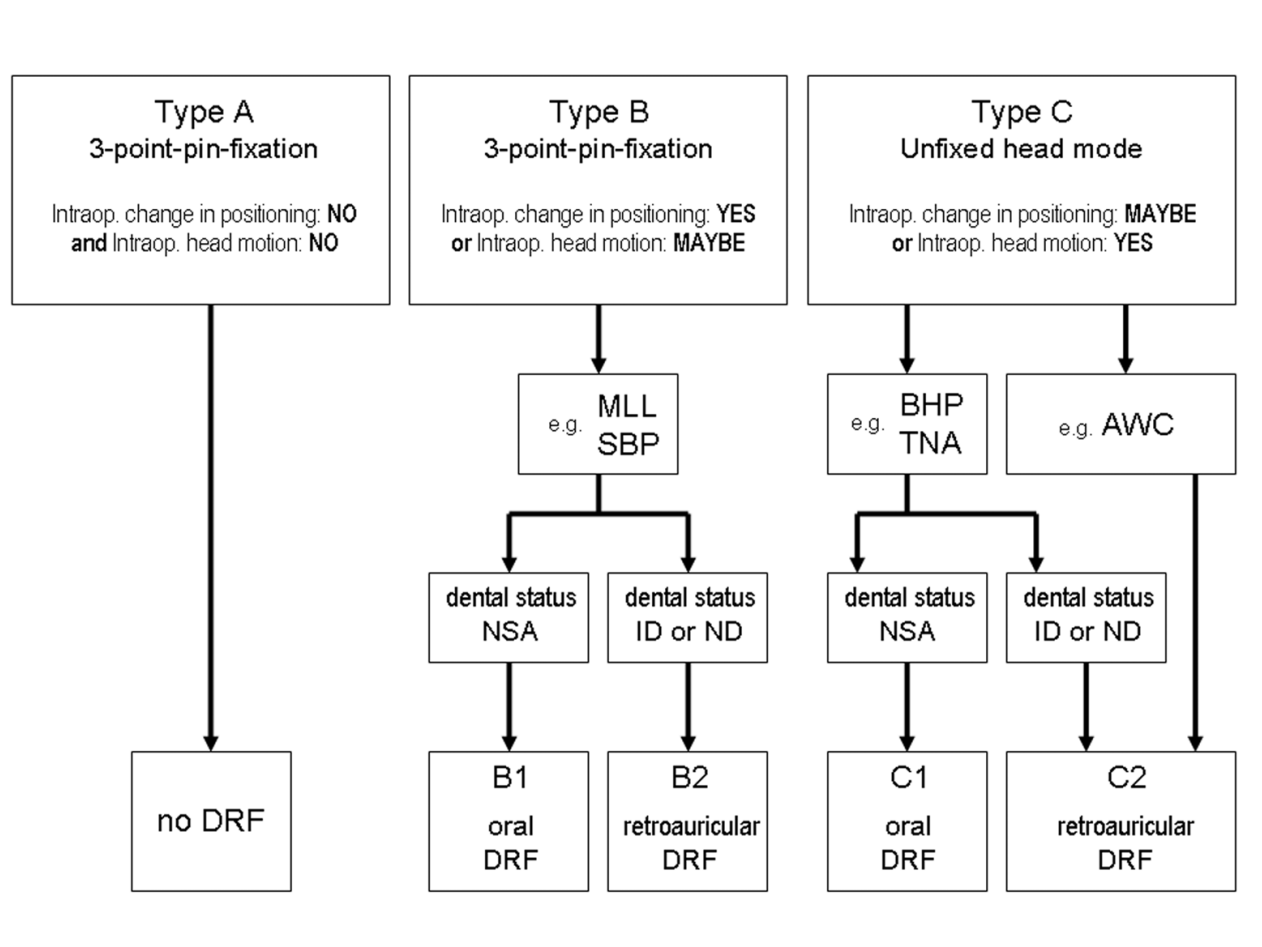
Types of intraoperative head fixation with and without DRF dependent on the diagnosis/indication for navigation and the dental status. MLL = Multilocular lesion, SBP = Skull base procedure, BHP = Burr hole procedure, TNA = Transnasal approach, AWC = Awake craniotomy, NSA = No significant abnormalities, ID = Inadequate dentition, ND = No dentures.

According to the indication for the use of intraoperative navigation, the patients were assigned to one of three types of interventions according to head fixation and use of the DRF including its mode of attachment (Figure 6). To assign the patients to one of the three groups, the following questions were answered: „Is it planned to reposition the patient/the patient's head during the operation?” and „Is it likely that there will be involuntary head movement during certain surgical manoeuvres?”

Type A comprised 10 patients in whom no intervention-related repositioning was planned and in whom involuntary movement of the head was unlikely because 3-point pin fixation was used. These patients were operated on with navigation performed under standard conditions and without additional use of the DRF. The patients of this group served as controls.

**Figure 7:**
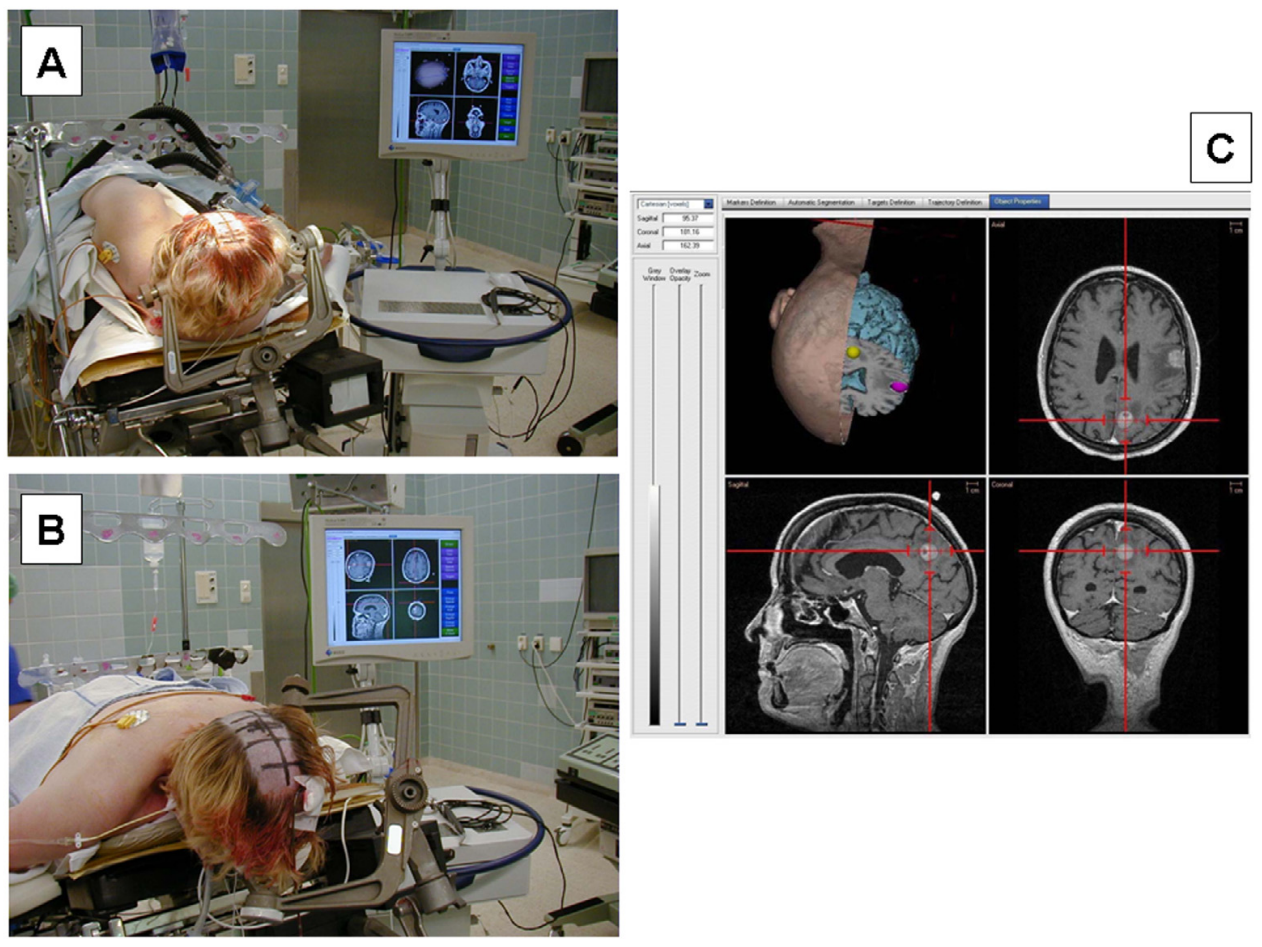
Type B_1 _fixation of the head. **(A) **Patient positioned on the right side for resection of a left frontal metastasis. **(B) **After repositioning in the prone position for resection of a left parieto-occipital metastasis. **(C) **Screenshot of the navigation system showing the location of the two tumours. 3p = Three point; r.a. = retroauricular.

Type B consisted of two subgroups. The first subgroup included those cases in whom intraoperative repositioning of the head was planned. These were 6 patients scheduled for removal of two lesions in one session. All 6 patients were actually repositioned during the operation. The other 4 patients assigned to this group had large lesions at the skull base, making it likely that voluntary or involuntary changes in head position would occur during the intervention. All 10 patients of this group were operated on using 3-point pin fixation in combination with a DRF. The DRF was attached orally (type B1) in 5 cases (Figure 7) and retroauricularly in the other 5 cases (type B2).

Type C consisted of those cases in whom intraoperative head movement was expected or desirable as well as those patients in whom repositioning might have become necessary in the course of the operation. These were 13 patients scheduled for burr hole procedures for neuroendoscopic interventions or biopsy, 3 patients in whom a transnasal approach was planned, and 4 patients undergoing awake craniotomy for removal of lesions from language areas. In 10 patients of this group, the DRF was attached in the oral cavity (type C1); in the other 10 cases, retroauricular attachment was necessary because of the dental status or for anesthesiological reasons and in the patients who underwent awake craniotomy to perform intraoperative speech testing (type C2).

### Registration accuracy

**Figure 8:**
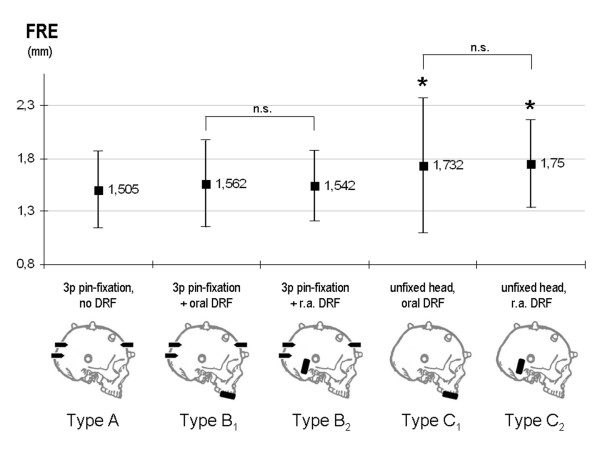
Fiducial Registration Error (FRE in mm) in the clinical application with head fixation types A, B1, B2, C1 and C2. Data are presented as mean +/- standard deviation (*n *= *5 patients in B1 and B2*; *n *= *10 patients in A*, *C1 and C2*). FREs in types C1 and C2 were significantly higher than in control group (type A) (* *p < 0,05, t test*). There was no statistical difference between the two B-type (B1 vs. B2) and the two C-type (C1 vs. C2) procedures (*n.s*. = *p *> 0.05)

The mean fiducial registration errors (FREs) were 1.51 mm (+/- 0.36 mm SD) in the control group type A (*n *= 10), 1.56 mm (+/- 0.40 mm SD) in type B1 interventions (*n *= 5), 1.54 mm (+/- 0.33 mm SD) in type B2 (*n *= 5), 1.73 mm (+/- 0.63 mm SD) in type C1 (*n *= 10), and 1.75 mm (+/- 0.41 mm SD) in type C2 (*n *= 10) (Figure 8). There was no statistically significant difference between rigid pin fixation (r.p.f.) of the head alone (control group: type A) and r.p.f. with additional DRF (type A vs. type B1; p > 0.05 and type A vs. type C2, p > 0.05). In DRF-supported procedures, there was no statistically significant difference between oral and retroauricular placement of the DRF, neither in cases with rigid pin fixation (type B1 vs. type B2, p > 0.05), nor in the unfixed head mode (type C1 vs. type C2, p > 0.05). However, both types of unfixed head positioning (type C1 and C2) presented with significant higher FRE mean values compared to control type A (type A vs. type C1; p < 0.05 and type A vs. type C2; p < 0.05), as well compared to both groups of r.p.f. with additional DRF (type C1 vs. B1, p < 0.05; type C1 vs. B2, p < 0.05; type C2 vs. type B1, p < 0.05 and type C2 vs. type B2, p < 0.05).

### Application accuracy

**Figure 9:**
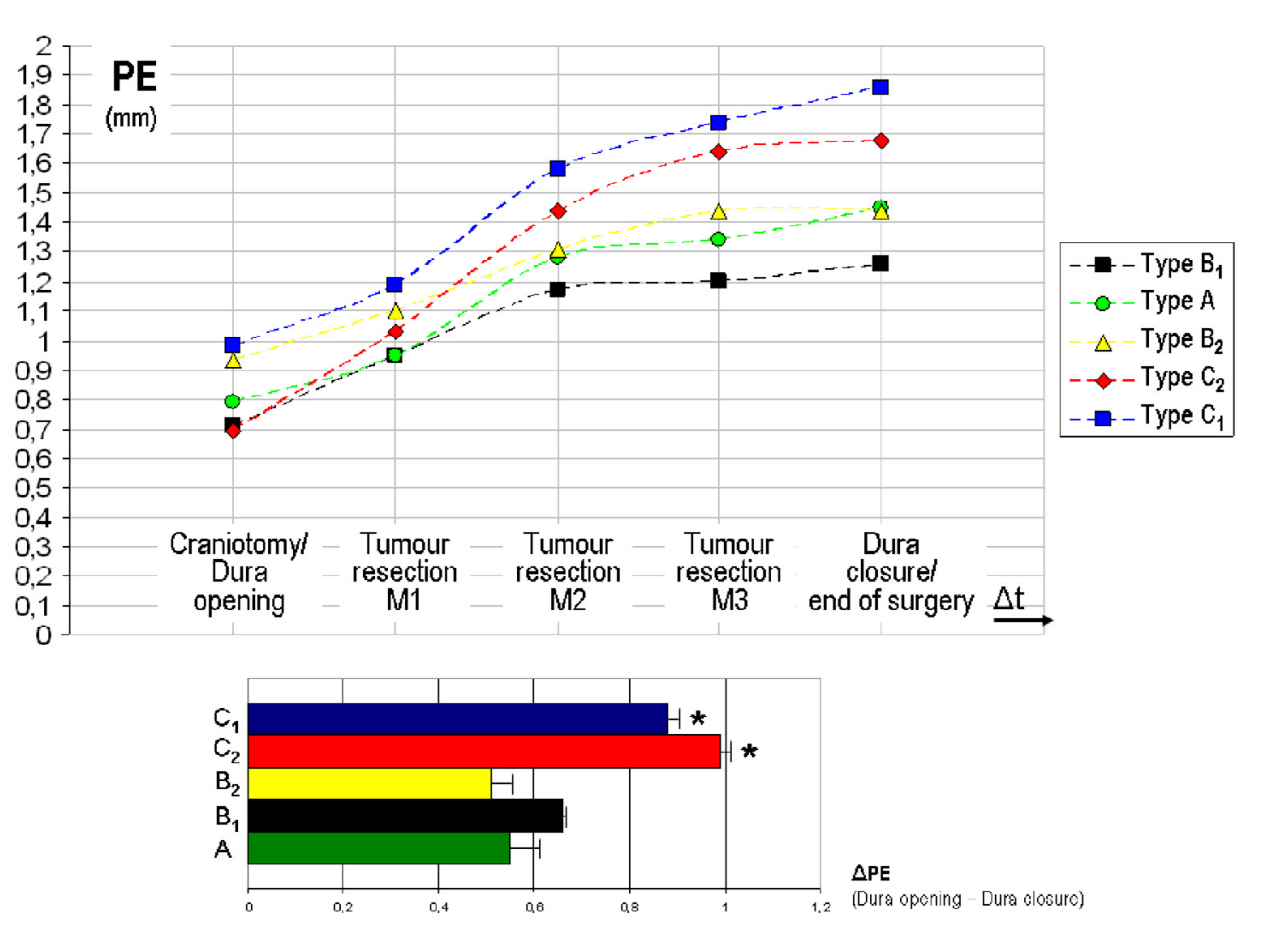
Mean position errors (PE in mm) in Type A-, B1-, B2-, C1- and C2- interventions measured after completion of craniotomy and before dura opening, at three consecutive time points (M1, M2 and M3) during tumour resection and after dura closure or at the end of the intervention. ΔPEs (measured as difference in mm between the initial PE at the time of dura opening and the corresponding PE at the time of dura closure) were significantly higher in types C1 and C2 compared with control group (types A) (* *p < 0,05, t test*). There were no statistical differences between the two B-type procedures and control group (type A) (*p *> 0.05).

The mean position errors (PEs) measured after completion of craniotomy and before dura opening (on average 71 min after the end of image data registration) were 0.79 mm (+/- 0.23 mm SD) in type A interventions, 0.71 mm (+/- 0.18 mm SD) in type B1, 0.93 mm (+/- 0.34 mm SD) in type B2, 0.98 mm (+/- 0.31 mm SD) in type C1, and 0.69 mm (+/- 0.25 mm SD) in type C2. The mean PEs measured at 3 consecutive time points during tumour resection (M1= about 20 min after dura opening/M2= about 45 min after dura opening/M3= about 70 min after dura opening) were 0.95/1.28/1.34 mm in type A, 0.95/1.17/1.20 mm in type B1, 1.10/1.31/1.44 mm in type B2, 1.19/1.58/1.74 mm in type C1, and 1.03/1.44/1.64 mm in type C2. The final measurements for determining application accuracy at the time of dura closure or at the end of the intervention (on average 84 min after dura opening) yielded mean PEs of 1.45 mm (+/- 0.34 mm SD) for type A interventions, 1.26 mm (+/- 0.29 mm SD) for type B1, 1.44 mm (+/- 0.30 mm SD) for type B2, 1.86 mm (+/- 0.29 mm SD) for type C1, and 1.68 mm (+/- 0.38 mm SD) for type C2 (Figure 9). ΔPEs (measured as difference in mm between the initial PE at the time of dura opening and the corresponding PE at the time of dura closure) were significantly higher in types C1 and C2 compared with control group (types A) (* *p < 0,05, t test*). There were no statistical differences between the two B-type procedures and control group (type A) (*p *> 0.05).

### Complications

Complications such as damage to the teeth or loosening resulting from oral attachment of the DRF were not observed in any of the 15 cases (types B1 and C1). Two patients showed small pressure sores on the lips due to direct contact with the plastic coating of the DRF. The sores healed spontaneously and without complications in the course of three days.

Retroauricular DRF attachment was also not associated with any major complications. None of the 15 patients in this group developed any pressure sores in the area of the auricle or the skin over the mastoid (types B2 and C2). The waterproof self-adhesive film prevented detachment of the tape, e.g. by disinfectant, in all 15 cases. One patient developed an allergic skin reaction to the tape used. The irritated skin responded well to topical ointment application and the irritation resolved within 24 hours.

## Discussion

Neuronavigation systems for frameless stereotaxy have been used in cranial neurosurgery since the middle of the 1980s [1,2]. Owing to the rapid technological development in the field of image processing and in computer technology, such navigation systems are now used routinely. They are helpful not only in intraoperative anatomical orientation, but also in delimitation of healthy from pathological processes and in achieving accurate alignment of instruments at the operation site. Although computer- and image-guided surgical procedures are an improvement of frame-guided stereotaxy, most navigation systems for frameless stereotaxy still require rigid fixation of the patient's head throughout the operation.

As with the referencing methods of other standard navigation systems [4,5], any change in the relative positions of the head and the transmitter used for reference leads to a loss of alignment between the virtual and the actual coordinate system in electromagnetic systems as well. Thus, a change in head position upon completion of image data registration precludes navigation unless the registration procedure is repeated. For these reasons, a second reference system is required that is attached directly to the target object in order to track all movements in relation to the primary coordinate system in those situations where movement cannot be excluded or rigid pin fixation of the patient is not desirable.

### Frameless stereotaxy without rigid-pin fixation of the head

Various techniques for identifying and compensating for intraoperative head movements during image-guided surgery have been published to date [12-18]. Ohhashi et al. [17], for example, reported on their experience with an electromagnetic navigation system originally developed for complex ENT operations of the sinus. This system comprises a plastic headset and does not require rigid head fixation. An advantage of this system described by its users is the fact that the headset simultaneously serves as a reference for automated image registration without the need for placement of fiducial markers. However, this system is geometrically restricted to the frontal region and the facial skull, ensuring an adequate navigational accuracy only in a limited volume around the headset, i.e. the area of the midface and the sinus. In contrast, the sensor-based DRF presented in this study enabled image data registration over the entire geometric area of the head where markers are placed. This is especially significant for intracranial operations as the number and size of the clusters registered directly affects the surgical accuracy in the respective area [9-11].

Another, really non-invasive attachment technique for a reference system, the so-called Vogele-Bale-Hohner mouthpiece, which moves with the patient's head was described by Bale and co-workers [18] in 2000. It acts as a reference arc that is attached to a tailored vacuum-affixed dental cast. The authors demonstrated that the accuracy achieved with their system is comparable to that of rigid pin fixation [18]. However, the size and shape of the reference frame that is also used for registration and the reliance on optical measurement also has drawbacks in that patient comfort is restricted and the system is of limited usefulness when the patient is positioned prone and the system thus comes to lie on the side away from the camera. Moreover, the device cannot be used in awake craniotomy with intraoperative language mapping since the mouthpiece does not allow the patient to speak. This problem can be overcome by the use of the sensor-based DRF and its positioning in the retroauricular area. Moreover, an electromagnetic system avoids the line-of-sight problem, i.e. it does not require an undisturbed visual contact between the DRF and a camera system. The here described electromagnetic technique thus allows unrestricted positioning as well as intraoperative repositioning of the patient. As the position of the sensor-bearing pointer is determined relative to the coordinate system defined by the DRF, the system operates in a virtual environment. Head movements and changes in position can be directly followed on the system's monitor in real-time. The same also holds true for image-guided instrument control. Here, very minute changes in position can directly affect the identification e.g. of a trajectory chosen on the basis of the image data.

### Accuracy of frameless stereotaxy with the DRF in the clinical application

The overall precision of the DRF in clinical use is affected by various factors. These include (a) the accuracy of the image data set employed, (b) various possibilities of error in the registration of the image data, (c) external effects on the positional accuracy, and (d) the technical accuracy of the pulsed DC magnetic measurement technique used:

(a) Both the voxel size and geometrical distortions in imaging (CT and MRI) have a direct effect on the accuracy of the 3D image data set. The maximum deviation that is possible corresponds to the extent of a voxel and thus crucially depends on the layer thickness of the tomographic technique used. This source of error is independent from the physical principle of the navigation technique.

(b) Various errors can be calculated as a measure for the quality of the image data registration. This is intended to enable comparisons between different navigation systems. In recent years, the nomenclature for analysis of measurement errors suggested by Fitzpatrick, Maurer and West [9,10] has become widely accepted. Usually, the fiducial registration error (FRE) is defined as a value for the measurement accuracy of image data registration. The FRE describes the distance between the position of a fiducial localized in the image data and the position measured in the operation site and transformed into the image coordinate system by means of the registration image. Important factors are the error in the positional measurement system used in determining the position in the operation site and the accuracy of the localization of the fiducial positions in the image data set. Statistical analysis of the FRE measurements in the patients of our study shows that there are no significant differences in mean registration accuracies between rigid 3-point pin fixation with (types B1 and B2) and without (type A) use of a DRF. This result suggests that there is no immediate benefit from the use of an additional DRF in terms of accuracy of image data registration. The registration accuracies in patients without rigid fixation of the head (types C1 and C2) did not differ significantly between oral and retroauricular attachment of the DRF. However, FRE values for types C1 and C2 were significantly higher (on average 10–15%) than in patients with rigid pin fixation. This may be caused by inadvertent micro movements of the Fiducial positions during the registration procedure, e.g. due to the effect of respiration on the unfixed head. Nevertheless, also C1 and C2 type values seem to be in an acceptable range for microneurosurgical procedures (means: C1 = 1.73 mm +/-SD; C2 = 1.75 mm +/- SD) as they compare well with those achieved with other standard navigation techniques since most commercial navigation systems assume registration to be successful if the FRE is less than 3 mm.

In order to detect a fall in application accuracy, e.g. due to displacement of the DRF, at an early stage and to be able to act on it, a fixed landmark can be checked at defined times over the entire period of the operation. As such a landmark, which is virtually undisplaceable, a burr hole near the craniotomy was chosen. To quantify this measure the so-called Position Error (PE) can be assessed. The PE describes the extent to which the accuracy of point localization changes between two or more time points in the course of the operation. The results listed in Figure 9 show that measuring accuracy in general decreases with the length of the operation. This is due to various factors such as the actual number of head movements having occurred at the time of measurement and physiological factors such as changes in skin turgor in the retroauricular cases. Additionally, the angle at which the stylus is held when pinpointing the 1 mm burr hole has a significant impact on measurement accuracy in the submillimeter range. This may explain the apparent improvement of the PE between two measurements in some of the cases. In general, our results demonstrate that the additional use of a DRF in combination with pin fixation of the head (types B1 and B2) has an application accuracy averaged over time which is comparable to that achieved with permanent rigid head fixation (control group: type A), even when head position is changed intraoperatively. Intraoperative changes in position are thus fully compensated for by use of the DRF. The fact that the lowest ΔPEs were obtained for type B2 interventions as compared with type A suggests that in type B2 operations even inadvertent displacement of the pin fixation has been compensated for. The significantly higher ΔPE values obtained in the group of patients operated on without rigid head fixation (types C1 and C2) are most likely attributable to a wider range of detectable movements, resulting in a higher degree of deterioration in the application accuracy over the time of surgery. However, mean ΔPE values for C-type procedures did not exceed 1 mm and over all PEs were less than 2 mm. The authors believe, that this makes intraoperative head tracking by DRF a suitable alternative to conventional rigid head fixation for certain indications, such as burr hole procedures, transnasal/transsphenoidal approaches and awake craniotomies. Oral attachment of the DRF was found to be superior to retroauricular attachment in combination with both types of head positioning (B:fixed and C:unfixed). Retroauricular attachment should therefore be reserved to those patients in whom oral fixation is not possible.

(c) Another critical issue is the potential interference of nearby conductive metals with the electromagnetic measuring technique. The susceptibility to such external distortion can be markedly reduced by using a DC technique instead of an AC technique [8]. The measuring accuracy of DC probes is nearly completely unaffected by most of the instruments of a neurosurgical standard set. Nevertheless, interferences occurs, for instance with the equipment used for intraoperative electrophysiological testing. This is why care must be taken when using the setup described here not to inadvertently induce an electrical field by performing electrical stimulation while the position of the cortical stimulation site is being determined by the navigation system. Due to the electromagnetic principle, the reference sensor may not be used with intraoperative MRI as well.

(d) The system accuracy of the electromagnetic navigation technique is essentially determined by the physical function principle as well as the hardware and software components implementing this principle. For the electromagnetic localizing sensor embedded in the DRF this is expressed technically by the term "static accuracy", i.e. by how accurately the position and the orientation of a sensor in space can be determined. The static accuracy (1.8 mm RMS – position and 0.5° RMS – orientation as specified by the manufacturer) is defined as root mean squared deviation of a true measurement of the magnetic centre of a single sensor with respect to the magnetic centre of a single transmitter measured over the translation range. Accuracy varies from one location to another over the translation range and will be degraded if there are interfering electromagnetic noise sources or metal in the operating environment.

### Fixation and attachment technique

The use of a dental splint attached to the upper row of teeth appears to be feasible in patients with healthy teeth. The self-hardening material used here is non-toxic, hypoallergic, fast-drying, and form-stable. Firm attachment is ensured as long as a vacuum is maintained between the hardened 2-component polyether system and the dental surface. Once the vacuum is released, e.g. with a small dental hook, the splint can be removed easily and rapidly. Others have described the ease of using such material intraoperatively. For example, Bale et al. [15,16,18] used a comparable polyether material for attachment of the mouthpiece of the Vogele-Bale-Hohner head holder. After two of our patients developed pressure sores on the lips, we have since placed a wet compress around the DRF following its placement. The compress protects the oral soft tissue and additionally prevents drying of the oral mucosa. No pressure sores have occurred thereafter.

Retroauricular attachment with adhesive tape was found to be inferior to oral attachment in terms of application accuracy, which is probably due to displacement of the DRF on the skin although no loosening of the tape was noted in any of the patients. Nevertheless, we think that a decrease in skin turgor and the weight of the sterile film are potential causes of the inaccuracies measured. With this potential source of inaccuracy in mind, one can still use retroauricular attachment of the DRF with benefit in patients with a poor dental status and in patients scheduled for awake craniotomy, as in patients undergoing awake craniotomy, pain associated with placement of the pins [20] and the risk of injuries through inadvertent head movements, less patient comfort, and declining ability of the awake patient to cooperate as the operation proceeds interfere with rigid fixation of the head.

## Conclusion

Rigid pin fixation of the head for microneurosurgical procedures in combination with frameless stereotaxy has to be considered gold standard, since highest accuracy is achieved only with intraoperative immobilization of the patient's head. However, based on the experience gained with intraoperative motion tracking, the authors see a high clinical potential for DRF application in cranial navigation. The use of an additional reference sensor increases the application scope of image-guided navigation procedures to include, for example, any bioptic or endoscopic intervention, in which rigid pin fixation of the cranium is not required or desired. The system allows highly flexible variation of the surgical strategy including intraoperative repositioning of the patient without impairment of navigational accuracy. In awake craniotomy patient comfort is improved by the fact that rigid pin fixation of the head is no longer required. For all other procedures, continuous tracking of head motion ensures automatic correction of spatial distortion with mechanical alteration of the head position. With a special dental cast for the oral attachment and the alternative option of a non-invasive retroauricular attachment, flexibility in the clinical use of the DRF is ensured.

## Abbreviations

AWC – Awake craniotomy

BHP – Burr hole procedure

CT – Computed tomography

DC – Direct current

DRF – Dynamic reference frame

FRE – Fiducial registration error

ID – Inadequate dentition

MLL – Multilocular lesion

MRI – Magnetic resonance imaging

ND – No dentures

NSA – No significant abnormalities

PE – Position error

r.a. – retroauricular

r.p.f. – rigid pin fixation

SBP – Skull base procedure

TNA – Transnasal approach

## Competing interests

The author(s) declare that they have no competing interests.

## Authors' contributions

OS – has defined conception and study design. He was responsible for collecting, analyzing and interpreting the data.

SS, SM, BK, SH, RS & TP – have made substantial contributions in collecting, analyzing and interpreting the data and have been involved in revising the manuscript critically for important intellectual content.

MB – has revised the manuscript critically for important intellectual content.

TK – has been involved in collecting and interpreting the data. He has revised the manuscript critically for important intellectual content and has given final approval of the manuscript to be published.

**Table 1:** Clinical data of 40 cases

**Patient No.**	**appl. type & procedure**	**gender & age**	**diagnosis**	**localization**	**CT/MRI-data**	**FRE (mm)**
1	A	m 28 y	glioma WHO III	left frontal	MRI	1,74
2	A	f 72 y	glioblastoma	left frontal	MRI	1,17
3	A	f 52 y	metastasis	right occipital	MRI	1,28
4	A	f 68 y	glioma WHO III	right parietal	MRI	2,16
5	A	f 81 y	glioblastoma	left parietal	MRI	1,57
6	A	f 56 y	metastasis	left temporal	MRI	1,65
7	A	f 73 y	metastasis	right frontal	MRI	1,36
8	A	m 30 y	metastasis	left temporal	MRI	0,91
9	A	m 61 y	glioblastoma	left parietal	MRI	1,37
10	A	m 60 y	glioblastoma	left parietal	MRI	1,84
						
11	B_1 _(MLL)	f 63 y	metastasis	left frontal/left parietal	MRI	2,11
12	B_1 _(MLL)	m 63 y	metastasis	right frontal/left temporal	MRI	1,24
13	B_1 _(MLL)	f 48 y	metastasis	left frontal/right frontal	MRI	1,85
14	B_1 _(MLL)	f 66 y	metastasis	left frontal/left occipital	MRI	1,44
15	B_1 _(SBP)	m 49 y	prim. bone tumour	skull base + orbita	CT	1,17
						
16	B_2 _(MLL)	f 67 y	metastasis	right frontal/right parietal	MRI	1,41
17	B_2 _(MLL)	f 68 y	metastasis	right frontal/right parietal	MRI	1,33
18	B_2 _(SBP)	m 54 y	meningioma	skull base + orbita	CT	1,24
19	B_2 _(SBP)	f 52 y	meningioma	skull base + orbita	CT	1,68
20	B_2 _(SBP)	m 67 y	prim. bone tumour	skull base + orbita	CT	2,05
						
21	C_1 _(BHP)	f 18 y	astrocytoma WHO II	left temporal	MRI	2,54
22	C_1 _(BHP)	m 56 y	astrocytoma WHO III	left temporal	MRI	1,87
23	C_1 _(BHP)	m 27 y	lymphoma	right parietal	MRI	1,57
24	C_1 _(BHP)	m 61 y	glioblastoma	left frontal	CT	1,21
25	C_1 _(BHP)	m 64 y	lymphoma	left temporal	MRI	1,17
26	C_1 _(BHP)	m 46 y	lymphoma	right temporal	MRI	1,30
27	C_1 _(BHP)	m 65 y	glioma WHO III	left temporal	MRI	2,98
28	C_1 _(TNA)	m 35 y	fibrous dysplasia	skull base	CT	1,32
29	C_1 _(TNA)	f 62 y	paraganglioma	right Fossa pterygopalatina	MRI	2.12
30	C_1 _(TNA)	m 54 y	fibrous dysplasia	skull base	CT	1,24
						
31	C_2 _(BHP)	m 64 y	lymphoma	left frontal	CT	1,33
32	C_2 _(BHP)	f 51 y	glioblastoma	left frontal	MRI	1,54
33	C_2 _(BHP)	m 54 y	astrocytoma WHO III	right frontal	MRI	1,87
34	C_2 _(BHP)	m 48 y	astrocytoma WHO II	left frontal	MRI	2,27
35	C_2 _(BHP)	m 65 y	metastasis	left frontal	CT	2,24
36	C_2 _(BHP)	f 70 y	glioblastoma	left temporal	MRI	1,56
37	C_2 _(AWC)	f 66 y	metastasis	left frontal	MRI	1,08
38	C_2 _(AWC)	m 26 y	glioma WHO III	left fronto-temporal	MRI	1,87
39	C_2 _(AWC)	f 63 y	glioma WHO III	left temporal	MRI	2,23
40	C_2 _(AWC)	m 56 y	glioma WHO III	left temporal	MRI	1,51

**m **= male; **f **= female; **y **= years; **FRE **= Fiducial Registration Error

**Type A **(control group) = 3-point-pin fixation; no DRF

**Type B**_1 _= 3-point-pin fixation; oral DRF; **Type B**_**2 **_= 3-point-pin fixation; retroauricular DRF

**Type C**_**1 **_= unfixed head; oral DRF; **Type C**_**2 **_= unfixed head; retroauricular DRF

**MLL **= multilocular lesion; **BHP **= burr hole procedure for biopsy/endoscopy; **SBP **= Skull base procedure; **TNA **= transnasal approach; **AWC **= awake craniotomy
